# Efficacy and Safety of Bcl-2 Inhibitor Venetoclax in Hematological Malignancy: A Systematic Review and Meta-Analysis of Clinical Trials

**DOI:** 10.3389/fphar.2019.00697

**Published:** 2019-06-21

**Authors:** Qingfang Li, Li Cheng, Kai Shen, Hongyu Jin, Hui Li, Yuan Cheng, Xuelei Ma

**Affiliations:** ^1^Department of Biotherapy, Cancer Center, State Key Laboratory of Biotherapy, West China Hospital, Sichuan University, Chengdu, China; ^2^State Key Laboratory of Oral Diseases & National Clinical Research Center for Oral Diseases & Department of Preventive Dentistry, West China Hospital of Stomatology, Sichuan University, Sichuan, China

**Keywords:** venetoclax, Bcl-2 inhibitor, hematological malignancy, deletion of chromosome 17p, chronic lymphocytic leukemia

## Abstract

**Background:** B-cell leukemia/lymphoma-2 (BCL-2) protein is an important part of apoptotic pathway, which is overexpressed in chronic lymphocytic leukemia cells, non-Hodgkin lymphoma cells, and myeloma cells. Venetoclax (ABT-199/GDC-0199) is a highly selective bioavailable inhibitor of BCL-2 protein, which is more effective and less valid against BCL-xL in BCL2-dependent leukemia and lymphoma cell.

**Method:** We searched PubMed database using retrieval keyword venetoclax, ABT-199, or GDC-0199 and investigated the data of the involved articles. Considering variability in different studies, the overall response rate was collected to assess the efficacy. Meanwhile, adverse events (AEs) were summarized, and event rates were calculated to assess the safety.

**Results:** When patients were treated with venetoclax monotherapy, the most common AEs were nausea, diarrhea, neutropenia, fatigue and thrombocytopenia, and neutropenia. In addition, thrombocytopenia, anemia, febrile neutropenia, and leukopenia appeared more common and are considered as the severe AEs (defined as grade ≥ 3 AEs). Although the occurrence of thrombocytopenia was relatively high, it was not the most severe type. When compared with patients treated with venetoclax and other drugs, nausea, diarrhea, and thrombocytopenia were still the most common AEs occurrence in the patients of all the grade. The overall event rate was 73%, which is quite satisfactory.

**Conclusion:** Venetoclax is a mild and efficient drug in treating advanced hematological malignancy, which can be specially fit for the chronic lymphocytic leukemia patients with del[17p], and AEs are well tolerable. Few tumor lysis syndrome occurred after taking the drugs. The AEs aroused by venetoclax, and the combination is well tolerable. Therefore, the use of venetoclax monotherapy or its combination with other therapies is desirable in hematological malignancy.

## Introduction

B-cell leukemia/lymphoma-2 (BCL2) protein is considered as a key pathogenesis, which is overexpressed in chronic lymphocytic leukemia (CLL), non-Hodgkin lymphoma (NHL), and myeloma (ML) cells (Cimmino et al., 2005; Souers et al., 2013; Touzeau et al., 2014) as an important regulator of apoptotic pathway. Moreover, BCL2 protein is closely related to chemoresistance in hematological tumors (Marschitz et al., 2000). CLL is a prevalent adult blood tumor, whose prognosis can be predicted by the quantitation of BCL protein (Robertson et al., 1996; Cimmino et al., 2005), while BCL2 prevents cells from apoptosis by insulating proapoptotic stimuli instead of promoting proliferation of cells itself (Borner, 2003; Willis et al., 2003).

BH3 mimetics, a novel agent, initiates apoptosis by releasing proapoptotic proteins after it binds and suppresses antiapoptotic proteins, which can prevent the biology of BCL-2 protein family (Certo et al., 2006). BH3-mimetic drug is an emerging antineoplastic agent *via* simulating cellular antiapoptotic pathway, whose function is also shared by BCL2 protein (Oltersdorf et al., 2005; van Delft et al., 2006; Green and Walczak 2013). Navitoclax (ABT-737) is the first BH3-mimetic inhibitor of BCL2, which has been assessed in clinical trials and reported to have promising effect in relapsed CLL patients and observed to have positive responses in 35% of these patients (Roberts et al., 2012; Rudin et al., 2012). After its reported favorable tolerance against small cell lung cancer and lymphoma, the significant decrease of blood platelet counting in mice treated with navitoclax was also observed (Oltersdorf et al., 2005). The dose-dependent drug-related thrombocytopenia limits the advanced use of the navitoclax, despite the rapid recovery of platelet count after the primary treatment (Mason et al., 2007).

Venetoclax (ABT-199/GDC-0199) is another highly selective, orally bioavailable inhibitor of B-cell leukemia/lymphoma-2 protein, which is even more efficient than navitoclax and less valid against BCL-xL in BCL2-dependent leukemia and lymphoma (Souers et al., 2013). Compared with navitoclax, venetoclax poses less impact on platelets both *in vivo* and *in vitro* (Souers et al., 2013). Venetoclax prevents growth of primary CLL cells and a part of NHL cell lines *in vitro*. Meanwhile, it also manifested a promising antitumor efficacy *in vivo* (Souers et al., 2013). These unique characteristics provide insight in the treatment of leukemia and NHL. Patients treated with venetoclax plus rituximab maintained a high overall response rate (ORR) of 86% and possessed acceptable adverse events (AEs) in patients with CLL or small lymphocytic lymphoma (SLL) (Seymour et al., 2017). Subsequently, venetoclax was applied solely in relapsed or refractory (R/R) CLL patients, which also achieved a high ORR with acceptable toxicity (Stilgenbauer et al., 2016). Patients diagnosed as R/R NHL were tolerated with venetoclax monotherapy within a safety dose (Davids et al., 2017). AEs are inevitable, although the drug is tolerant. The M12-175 study is the first study in which R/R CLL or NHL patients received incremental dose of venetoclax. With gradual dose escalation, the risk of tumor lysis syndrome (TLS) increased (Roberts et al., 2016). Gradually, venetoclax combined with other drugs was also applied on multiple myeloma (MM) (Touzeau et al., 2014) and acute myelocytic leukemia (AML) (Konopleva et al., 2016; Wei et al., 2019). In this study, we analyzed these clinical trials to assess the safety and efficacy of venetoclax.

## Methods

### Study Design, Search Strategy, and Study Selection

Our research followed the guidance of the preferred reporting items for systematic reviews and meta-analysis statement. And the research question was structured according to the population, intervention, comparison, outcome format rules—population: hematological tumor adult patients; intervention: treated with BCL-2 inhibitor venetoclax (ABT-199 or GDC-0199); comparison: with/without control (patients treated with other drugs); outcomes: AEs after using the drug or the survival condition after using the drug, such as ORR, overall survival (OS), and progression free survival (PFS). A relevant literature search was performed on PubMed to identify relevant studies (up to May 2019). The following key words and their combinations were used without using any automatic filters: “venetoclax or ABT-199 or GDC0199.” In addition, the references of selected studies were also screened for additional relevant studies.

### Inclusion Criteria and Excluded Criteria

The eligibility criteria in the study were as follows: 1) clinical trials in any phase of venetoclax with the survival of the hematological malignancy patients or AEs reported in articles; 2) the patients enrolled in the trials who were suffering from hematological tumor were confirmed by pathology, regardless of any treatment before; 3) full data of the safety or efficacy were available in the articles; 4) full texts could be found; 5) the drug was applied on human; 6) patients’ age was over 18 years. Articles were excluded when no raw data were provided or data were duplicated.

### Data Extraction

Extracted data were as follows: 1) the fundamental information of studies: the first author name, ClinicalTrials.gov number, the phase of the articles, the year the articles were published, number of participating patients, cancer type, follow-up time, and age; 2) the characteristics of primary AEs were mentioned in at least two articles or in basic research articles. They are divided into AEs of all grades and grade ≥ 3 AEs; 3) Survival indicators, such as PFS, OS, or ORR of the patients.

### Statistical Analysis

The Comprehensive Meta-Analysis program 2 (Biostat, Englewood, NJ) was used to carry out the analysis of the survival and AEs data. We calculated the proportion and derived 95% confidence interval (CI) of major AEs (involving both all grade and grade ≥ 3) in included studies. When the two-sided *P* values were less than 0.10, they were regarded as significant. If *I* ^2^ ≥ 50%, it was considered as random-effects model in the analysis or else a fixed-effects model was applied.

### Quality of Studies

The systematic bias of the involved randomized controlled trial articles was evaluated by using the Cochrane’s risk of bias tool (Review Manager 5.3). The non-randomized trials were evaluated by the methodological index for non-randomized studies (Slim et al., 2003). These items are scored 0, 1, or 2. Score 0 demonstrates that the studies did not report this part; score 2 demonstrated the studies reported and were adequate. Score 1 demonstrates reported but not adequate. The global ideal score being 16 for studies that were non-comparative studies. Two researches scored each trial for the risk of bias independently. All the disagreement was solved by discussion.

## Results

### Study Selection and Characteristics

**Figure 1:**
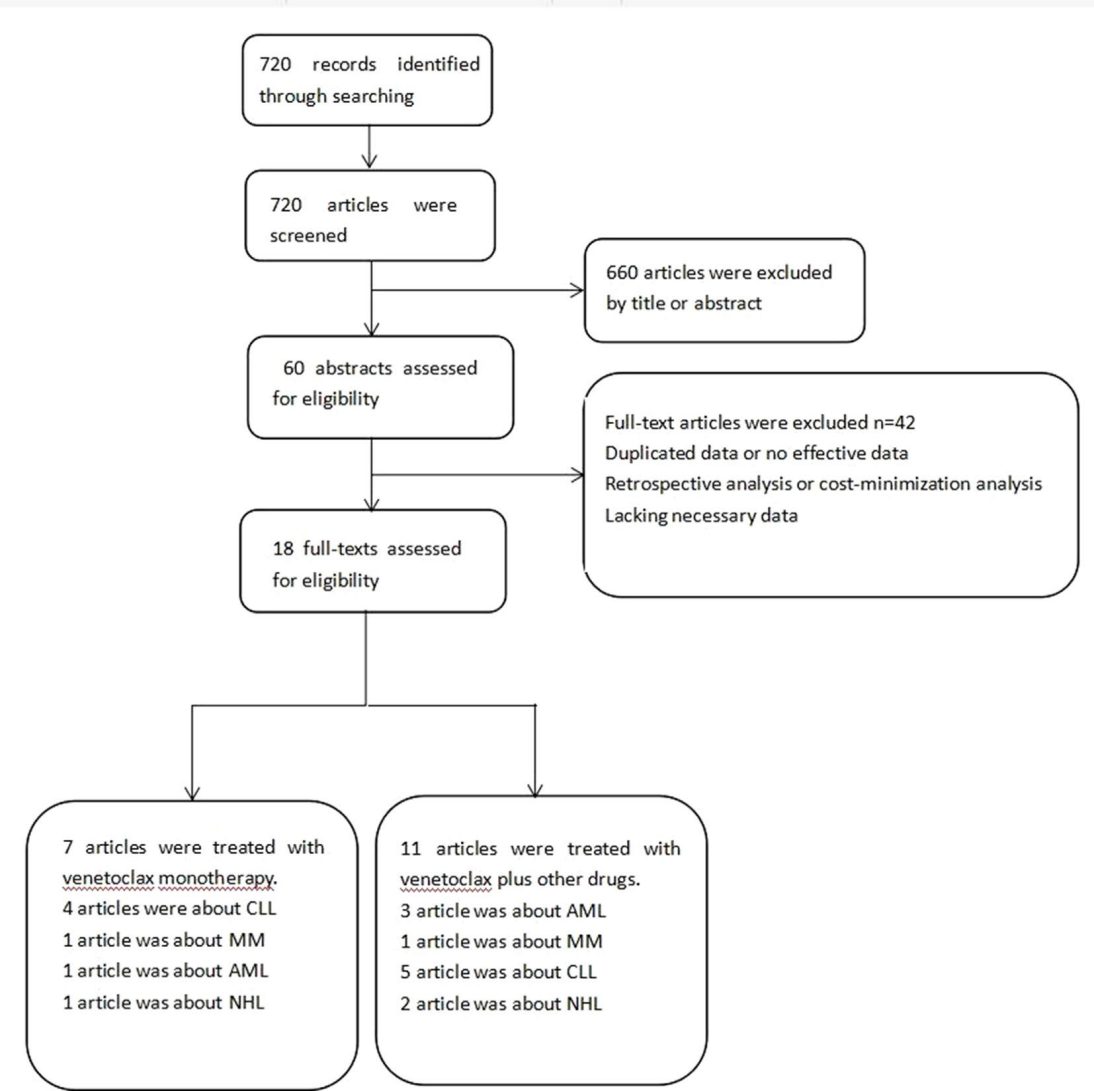
Flow diagram of the literature search and trial selection process.

Seven hundred twenty possibly related articles were identified through searching in PubMed in May 2019. After carefully screening the titles and the offered abstracts, 41 articles were further investigated. Finally, 18 articles were involved in the research. Venetoclax monotherapy was reported in seven articles. The treatment used in the other 11 articles was venetoclax plus other therapies (Moreau et al., 2017; Seymour et al., 2017; Aldoss et al., 2018; Cramer et al., 2018; de Vos et al., 2018; DiNardo et al., 2018; Rogers et al., 2018; Flinn et al., 2019; Kater et al., 2019; Wei et al., 2019; Zelenetz et al., 2019). The details of the selecting process were shown in **Figure 1**. The basic information of the articles is listed in **Table 1**. Venetoclax and decitabine (group a), venetoclax and azacytidine (group b), or venetoclax, combined with decitabine and posaconazole (group c), were used by the patients in the study of DiNardo CD (DiNardo et al., 2018). Therefore, the involved patients were analyzed in three groups during the data analysis.

**Table 1:** Basic information.

Author	ClinicalTrials.gov number	Phase	Year	Number of patients	Study design	Cancer type	Follow-up	Age	MINORS score
Shaji Kumar	NCT01794520	1	2018	66	Single-arm	Relapsed or refractory *t*(11;14) multiple myeloma	Ongoing	63 years (31–79)	16
Matthew S. Davids	NCT01328626	1	2017	106	Single-arm	Relapsed or refractory non-Hodgkin lymphoma (mantle cell lymphoma (MCL; *n* = 28), follicular lymphoma (FL; *n* = 29), diffuse large B-cell lymphoma (DLBCL; *n* = 34), DLBCL arising from chronic lymphocytic leukemia (Richter transformation; *n* = 7), Waldenstro¨m macroglobulinemia (*n* = 4), and marginal zone lymphoma (*n* = 3))	5.3 months (0.2–46)	66 years (25–86)	13
Steven Coutre	NCT02141282	2	2018	36	Single-arm	Relapsed or refractory chronic lymphocytic leukemia	14 months (1–29)	68 years (56–85)	16
Jeffrey A. Jones	NCT02141282	2	2017	91	Single-arm	Relapsed or refractory chronic lymphocytic leukemia	14 months (8–18)	66 years (28–81)	16
Stephan Stilgenbauer	NCT01889186	2	2016	107	Single-arm	Relapsed or refractory chronic lymphocytic leukemia	12.1 months (10.1–14.2)	67 years (37–85)	16
Andrew W. Roberts	NCT01328626	1	2016	116	Single-arm	Relapsed or refractory chronic lymphocytic leukemia	17 months (1–44)	66 years (36–86)	16
Marina Konopleva	NCT01994837	2	2016	32	Single-arm	Relapsed or refractory acute myelogenous leukemia	Not given	71 years (19–84)	16
Philippe Moreau	NCT01794507	1b	2018	66	Single-arm	Relapsed or refractory multiple myeloma	5.9 months (0.3–29)	63 years (31–79)	16
Courtney D. DiNardo	NCT02203773	1b	2018	57	Single-arm	Untreated acute myeloid leukemia	12.4 months (8.3–15.8)	75 years (71–80)	16
Prof John F. Seymour	NCT01682616	1b	2018	49	Single-arm	Relapsed or refractory chronic lymphocytic leukemia	28 months (19–32)	68 years (50–88)	16
Andrew H. Wei	NCT02287233	1b/2	2019	82	Single-arm	Untreated acute myeloid leukemia	4.2 months (0.2–29)	74 years (63–90)	16
Andrew D. Zelenetz	NCT02055820	1b/2	2019	56	Single-arm	Non-Hodgkin lymphoma	22 months (11.4–36.4) Treatment duration	arm A 60.3 years (37–79); arm B 61.1 years (24–76)	14
Kerry A. Rogers	NCT02427451	1b	2018	12	Single-arm	Relapsed or refractory chronic lymphocytic leukemia	24.4 months (no mention)	57 years (42–70)	15
Sven de Vos	NCT01594229	1b	2018	60	Single-arm	Relapsed or refractory non-Hodgkin lymphoma	7.4 months (0.1–55.1)	62 years (29–90)	15
Ibrahim Aldoss	No mention	Respective analyze	2018	33	Single-arm	Relapsed or refractory acute myeloid leukemia	6.5 months (0.8–12.4)	62 years (19–81)	11
Ian W. Flinn	NCT01685892	1b	2019	77	Single-arm	Chronic lymphocytic leukemia	29.3 months (3–55) in R/R patients; 26.7 months (16–39) in 1L patients	61 years (42–80) in R/R patients; 63 in 1L patients (47–73)	16
Paula Cramer	NCT02401503	2	2018	66	Single-arm	Chronic lymphocytic leukemia	16 months (15–18)	59 years (54–67)	16
Arnon P. Kater	NCT02005471	3	2018	194	Randomized study	Relapsed or refractory chronic lymphocytic leukemia	9.9 months (1.4–22.5)	No mention	RCT

The study of Andrew W. Roberts and Matthew S. Davids share the same ClinicalTrials.gov number, but the patients reported in the studies were different. Andrew W. Roberts reported the relapsed or refractory chronic lymphocytic leukemia patients. Matthew S. Davids reported the relapsed or refractory non-Hodgkin lymphoma patients. In Andrew D. Zelenetz’s study, arm A is treated with venetoclax combined with R-CHOP, arm B is treated with venetoclax combined with G-CHOP. RCT, randomized controlled trial.

### Safety

**Figure 2:**
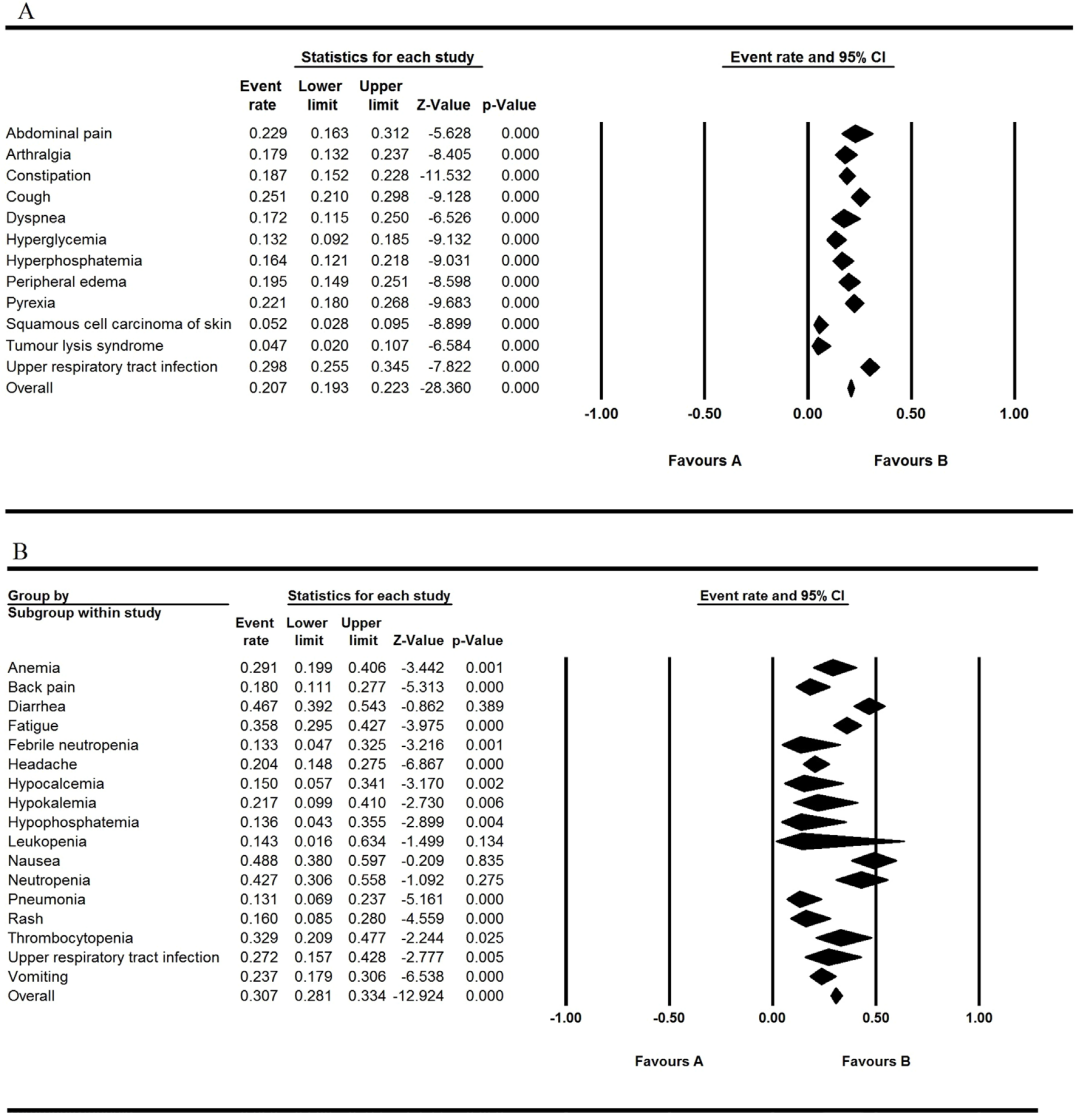
Result of all grade adverse events of venetoclax monotherapy. **(A)** Shows the fixed model and **(B)** shows the random model.

**Figure 3:**
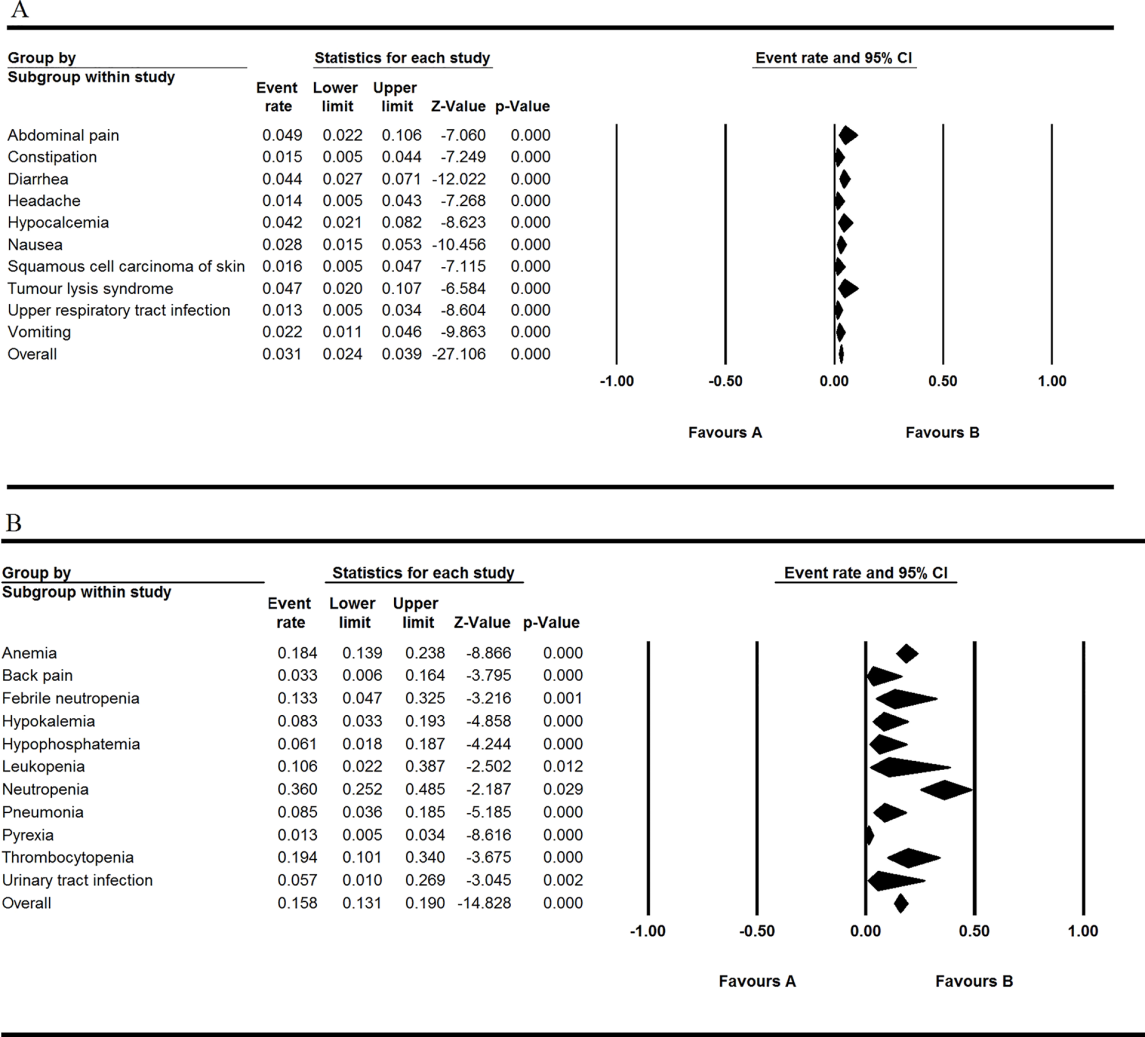
Result of the grade 3 or 4 adverse events of venetoclax monotherapy. **(A)** Shows the fixed model and **(B)** shows the random model.

**Figure 4:**
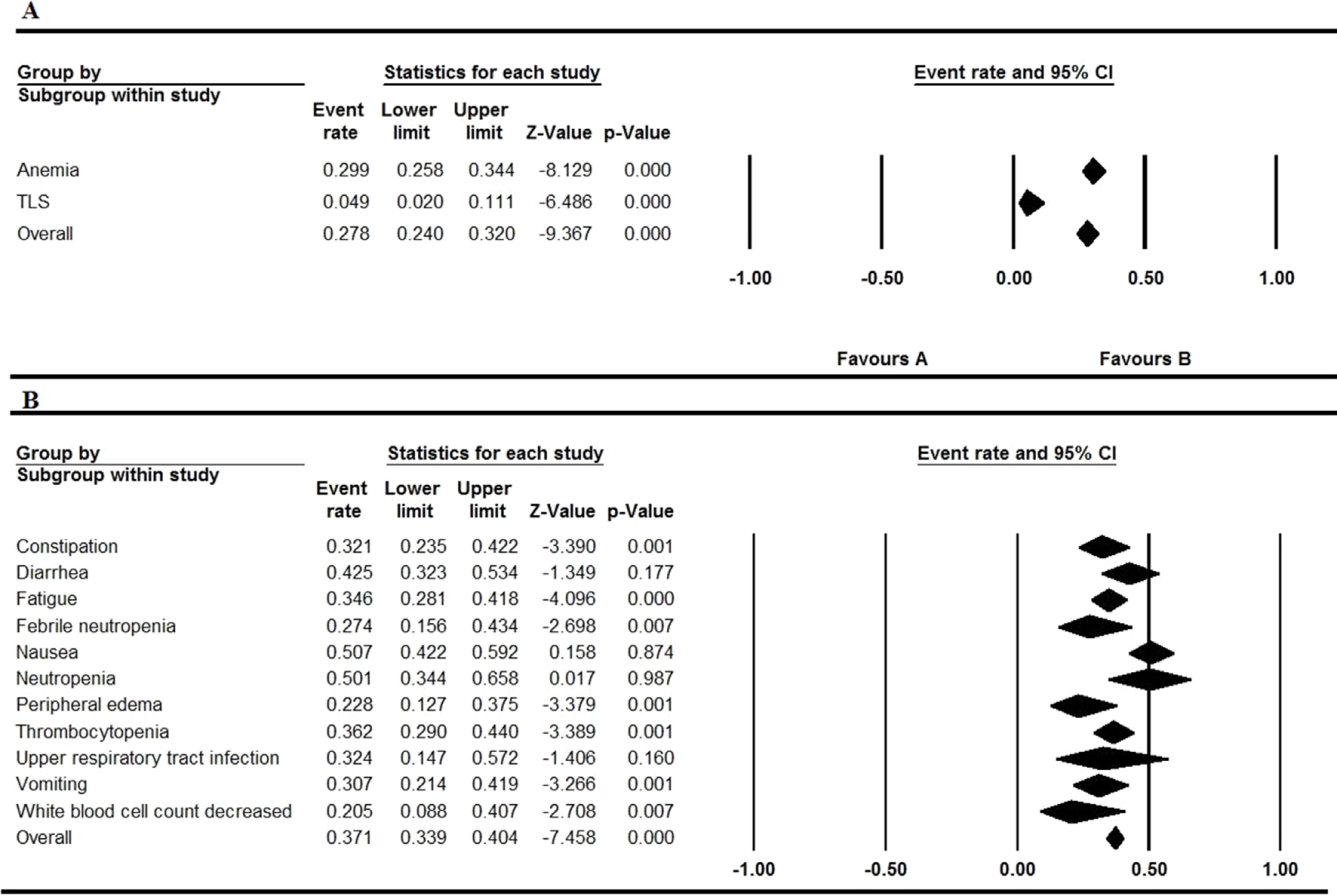
Result of all grade adverse eventsof venetoclax plus other drugs. **(A)** Shows the fixed model and **(B)** shows the random model.

**Figure 5:**
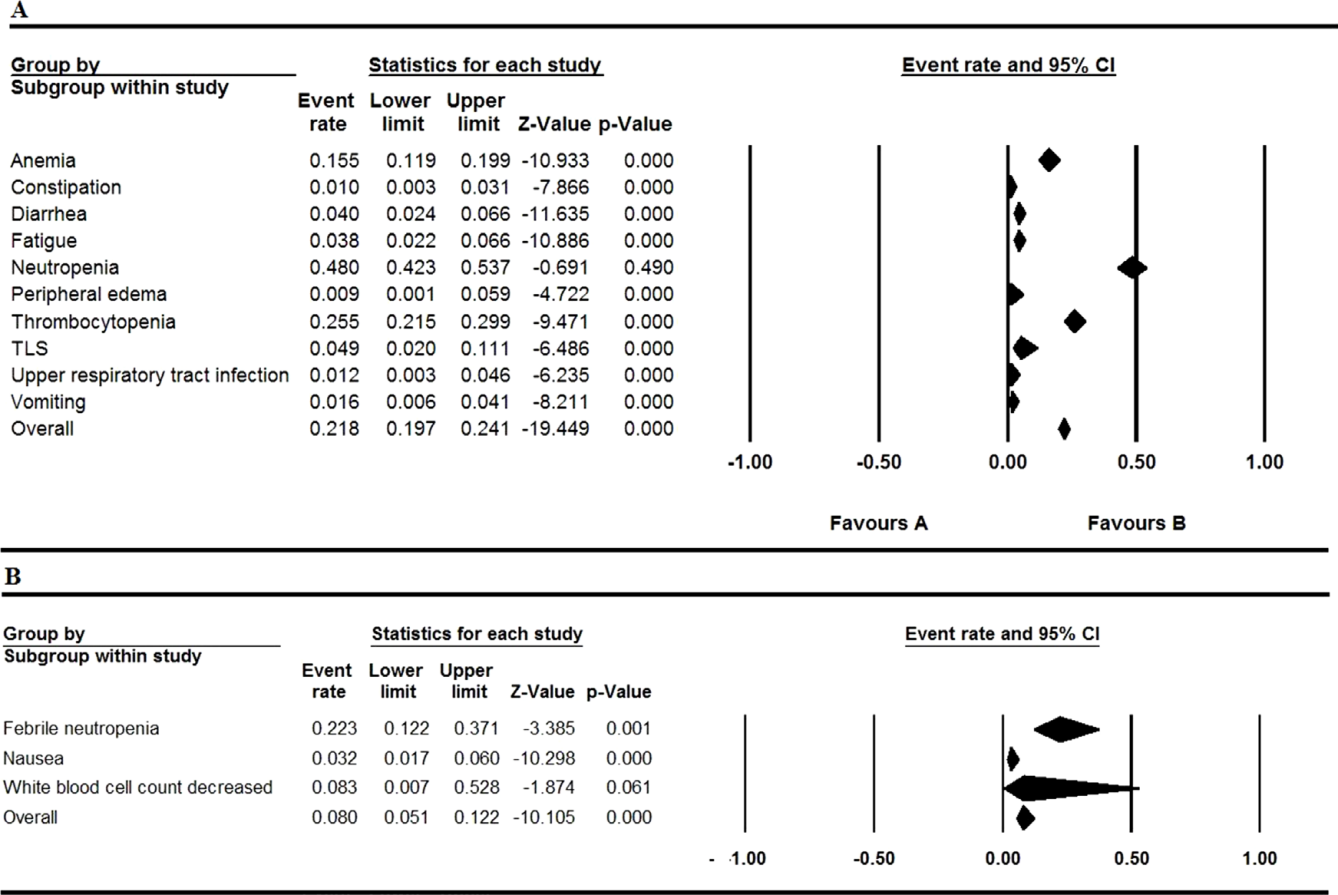
Result of the grade 3 or 4 adverse events of venetoclax plus other drugs. **(A)** Shows the fixed model and **(B)** shows the random model.

All the involved articles were single arms and reported AEs, so all the articles were used to calculate the AEs rate. The results were presented in **Figures 2** to **5**. **Figure 2** shows the result of all grade AEs of venetoclax monotherapy (fixed model A, random model B). **Figure 3** shows the results of the grade 3 or 4 AEs of venetoclax monotherapy (fixed model A, random model B). **Figure 4** shows the results of all grade AEs of venetoclax combination with other therapies (fixed model A, random model B). **Figure 5** shows the results of the grade 3 or 4 AEs of venetoclax combination with other therapies (fixed model A, random model B). **Table 2** shows the top five most frequently occurred AEs in venetoclax monotherapy.

**Table 2:** The top five most common AEs of all the grades and Grade ≥ 3.

AEs	Any grade				AEs	Grade ≥ 3			
	Event rate (95% CI)	*Z* value	*P* value	Statistical method		Event rate (95% CI)	*Z* value	*P* value	Statistical method
Nausea	0.488 (0.380–0.597)	–0.209	0.835	Random	Neutropenia	0.360 (0.252–0.485)	–2.187	0.029	Random
Diarrhea	0.467 (0.392–0.543)	–0.862	0.389	Random	Thrombocytopenia	0.194 (0.101–0.340)	–3.675	0.000	Random
Neutropenia	0.427 (0.306–0.558)	–1.092	0.275	Random	Anemia	0.184 (0.139–0.238)	–8.866	0.000	Random
Fatigue	0.358 (0.295–0.427)	–3.975	0.000	Random	Febrile neutropenia	0.133 (0.047–0.325)	–3.216	0.001	Random
Thrombocytopenia	0.329 (0.209–0.477)	–2.244	0.025	Random	Leukopenia	0.106 (0.022–0.387)	–2.502	0.012	Random

### Venetoclax Monotherapy

After evaluating all the AEs of venetoclax monotherapy patients, nausea, diarrhea, neutropenia, fatigue, and thrombocytopenia were found to be the top five most common AEs of all the grades, and neutropenia, thrombocytopenia, anemia, febrile neutropenia, and leukopenia were the most common AEs of grade ≥ 3 (severe AEs; these results are exhibited in **Table 2**). Of all the reported AEs of monotherapy, the occurrence of thrombocytopenia was relatively high, but it was not the most severe type. Five articles reported the incidence of thrombocytopenia ranging from 0.187 to 0.485, the overall event rate was 0.329 (95% CI 0.209, 0.477), and the grade ≥ 3 (severe AEs) event rate was 0.194 (95% CI 0.101–0.340). Nausea was the most common AE with the highest occurrence rate of 0.488 (95% CI 0.380–0.597), whereas the rate of neutropenia was 0.360 (95% CI 0.252–0.480), which is the most severe AE. Diarrhea and neutropenia were also the frequently reported AEs. With regard to single-arm trial monotherapy, the overall event rate of fatigue was 0.291 (95% CI 0.199–0.406). Despite the rate of neutropenia that reached to 0.360 (95% CI 0.252–0.485), other severe AEs (grade ≥ 3) were relatively rare.

### Venetoclax Combination With Other Therapies

Nausea, diarrhea, neutropenia, and thrombocytopenia were still the most common AE occurrence in the patients of all the grades through the analysis. Despite the high rates of neutropenia in 0.480 (95% CI 0.423–0.537), other grade ≥ 3 AEs (severe AEs) were relatively rare. Thrombocytopenia and diarrhea also maintain the overall event rate over 30% of all grades. Upper respiratory tract infection of all grades has the overall event rate of 0.324 (95% CI 0.147–0.572). The overall rate of grade ≥ 3 AEs (severe AEs) is lower than 10% except thrombocytopenia and anemia.

## Efficacy

### Venetoclax Monotherapy

**Figure 6:**
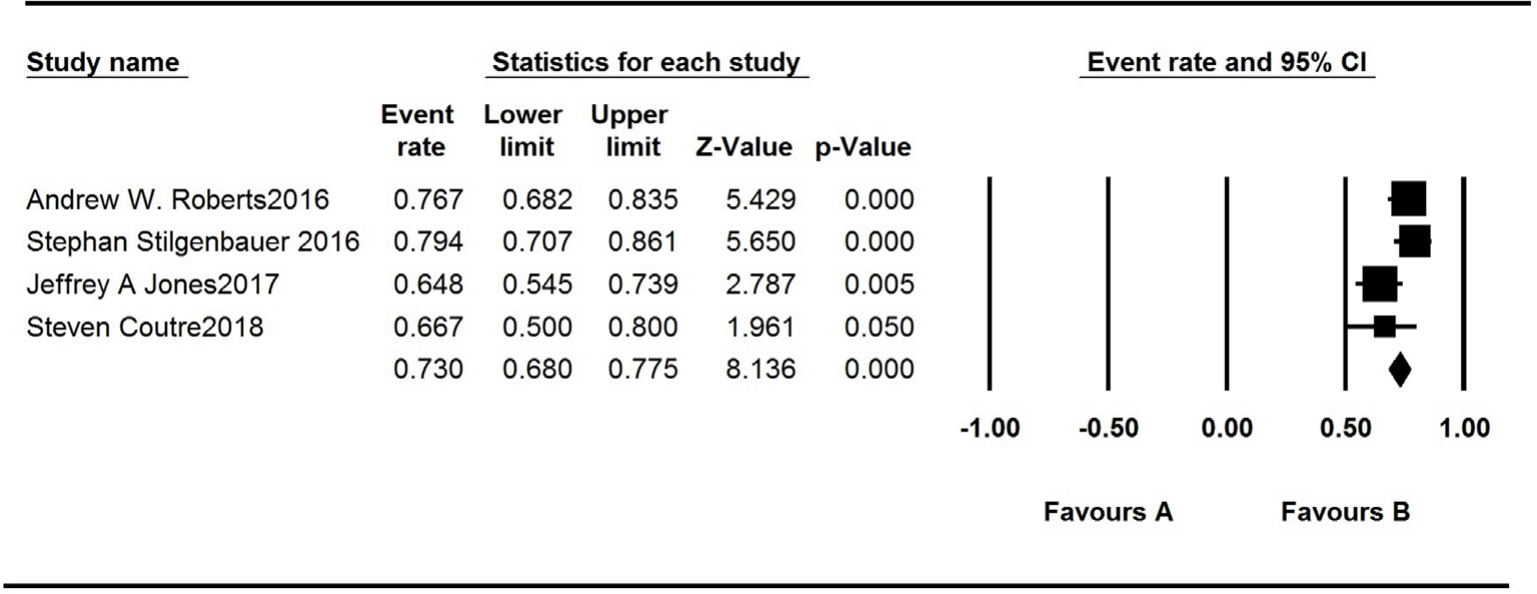
Analysis of the overall response rate of the studies.

There are seven articles concerning venetoclax monotherapy for treating CLL, MM, acute myelogenous leukemia, and NHL. The patients’ monotherapy survival rates recorded in the articles are listed in **Table 3**. PFS is approximately 25 months in studies by Roberts AW and Jones JA. The median first response time of CLL patients in four studies varied greatly. Two studies were 2.5 months, one was 6 weeks, and the other one is 0.8 month. The ORR of the AML patients was 19%, while that among *t* (11:14) MM patients was 40%. In patients who had been diagnosed as NHL, the ORR was 44%. The highest ORR was reported in patients with mantle cell lymphoma (MCL), which is 75%. The ORR of definite R/R CLL patients ranged from 64.8% to 79.4%, and the overall event rate was 73% (**Figure 6**), which is quite satisfactory.

**Table 3:** The monotherapy survival rates recorded in articles on patients.

Study	Year	Media first response	Media PFS	Media follow-up	ORR
Roberts AW	2016	6 weeks (5–24)	25 months (17–30)	17 months (1–44)	79%
Coutre S	2018	2.5 months (1.6–8.1)	NM	14 months (1–29)	67%
Jones JA	2018	2.5 months (1.6–2.6)	24.7 months (19.2–NT)	14 months (8–18)	65%
Stilgenbauer S	2016	0.8 months (0.7–1.7)	6.3 months (4.8–9.8)	12.1 months (10.1–14.2)	79.4%
Kumar S	2017	9.7 months (7.0–NT)	2.6 months (1.9–4.7)	2.5 months (0.2–25)	21%
Konopleva M	2016	144.5 days (83–256)	2.5 months (1–3)	63.5 days (14–256)	19%
Davids MS	2017	42 days (30–366)	6 months (4–10)	5.3 months (0.2–46.0)	44%

NT, not reached; PFS, progression-free survival; NM, not mentioned; ORR, overall response rate.

### Venetoclax Combination With Other Therapies

Survival data of the venetoclax plus other drugs are listed in **Table 4** (the OS and PFS are listed in **Table S1**). The response rate of R/R CLL patients treated by venetoclax plus rituximab was 82%. The ORR of the untreated AML patients was 64%, especially in the group of venetoclax plus decitabine and posaconazole in which the ORR was 67%, while that in the group venetoclax plus azacytidine was 59%. The ORR of the R/R MM patients was 67%. When CLL patients treated with venetoclax plus obinutuzumab, the ORR was high to 95%, and even all the untreated patients were in complete remission. According to Arnon P. Kater’s study, both PFS and OS of the venetoclax–rituximab arm were superior to the bendamustine–rituximab arm. Three year PFS of patients treated with venetoclax combined rituximab was 71.4%.

**Table 4:** Survival data of the venetoclax plus other drugs.

Study	Treatment	Disease	ORR
Philippe Moreau	Venetoclax plus bortezomib and dexamethasone	Relapsed or refractory multiple myeloma	67%
Prof John F Seymour	Venetoclax plus rituximab	Relapsed or refractory chronic lymphocytic leukemia	82%*
Courtney D DiNardo A	Venetoclax plus decitabine	Untreated acute myeloid leukemia	65%
Courtney D DiNardo B	Venetoclax plus azacitidine	Untreated acute myeloid leukemia	59%
Courtney D DiNardo C	Venetoclax plus decitabine and posaconazole	Untreated acute myeloid leukemia	67%
Courtney D DiNardo		Untreated acute myeloid leukemia	64%
Ibrahim Aldoss	Venetoclax plus decitabine or 5-azacitidine	Relapsed or refractory acute myeloid leukemia	64%
Paula Cramer	Venetoclax plus obinutuzumab	Chronic lymphocytic leukemia	95%
Andrew H.Wei	Venetoclax plus cytarabine	Untreated acute myeloid leukemia	54% (CR)
Kerry A. Rogers	Venetoclax plus obinutuzumab and ibrutinib	Relapsed or refractory chronic lymphocytic leukemia	92%
S. de Vos	Venetoclax plus bendamustine and rituximab	Relapsed or refractory non-Hodgkin lymphoma	65%
Andrew D. Zelenetz	Ventoclax plus R/G-CHOP	Non-Hodgkin lymphoma	87.5%
Ian W. Flinn	Venetoclax plus obinutuzumab	Chronic lymphocytic leukemia	95% (R/R), 100 (1L)
Arnon P. Kater	Venetoclax plus rituximab	Relapsed or refractory chronic lymphocytic leukemia	NM

ORR, overall response rate; NM, not mentioned; CR, complete remission. *This article provided the rate of response.

### Assessment of Study Quality and Publication Bias

Review Manager 5.3 was used to assess the methodological quality of all the involved studies. **Figures S1** and **S2** show the risk of bias graph and risk of bias summary of the researches. The non-randomized studies were assessed by the methodological index for non-randomized studies score; most of the studies get a satisfactory score (**Table 1**), except a retrospect study that gets 10.

## Discussion

Venetoclax is a highly selective oral BCL2 inhibitor with high sensitivity in patients with CLL/SLL, including patients who were treated with first-line and second-line chemotherapy or having 17p deletion (Levy and Claxton, 2017). As the first BCL-2 inhibitor approved by the US Food and Drug Administration, BCL-2 inhibitor was effective in CLL and recommended for R/R CLL after being treated by ibrutinib according to National Comprehensive Cancer Network. It was also regarded as the second-line medicine in MCL patients. Clinical trials of venetoclax in ER- and BCL2-positive metastatic breast cancer were reported (Lok et al., 2019), and BCL-2 is a potential target in treating nasopharyngeal carcinoma (Wang et al., 2018) and small cell lung cancer (Inoue-Yamauchi et al., 2017; Lam et al., 2017; Lochmann et al., 2018). This study investigated the efficacy and safety of venetoclax in hematological malignancies.

With regard to the safety, we analyzed the AEs of patients taking venetoclax orally. The most common AEs were diarrhea, nausea, neutropenia, fatigue, thrombocytopenia, anemia, cough, pyrexia, and abdominal pain. Nausea occurred in almost half of the patients, while these patients rarely experienced severe nausea. Severe AEs are relatively rare except for neutropenia among monotherapy patients. Thrombocytopenia and anemia were the most severe AEs among the venetoclax plus other therapies. Notably, hematologic toxicities were the most frequent severe AE. The ORR of the monotherapy patients was surprisingly encouraging, with three quarters of the patients benefiting.

TLS is an important toxic effect in the first-in-human phase 1 study in patients who had been diagnosed as R/R CLL or SLL (Roberts et al., 2016). The risk of TLS has relevance with the rapid CLL cells reduction, which can be alleviated by gradual dose escalation therapy (Howard et al., 2011; Souers et al., 2013; King et al., 2017). The safety evaluation of some other clinical trials suggested initiating with 20-mg venetoclax for 1 week followed by no additional intensive prophylactic measures (Roberts et al., 2016). Comparatively, TLS rarely happened to CLL patients in this therapeutic scheme even in venetoclax combined with other drugs (Coutre et al., 2018; Jones et al., 2018). Except for CLL patients, TLS events were not observed in MM, NHL, or AML patients (Konopleva et al., 2016; Davids et al., 2017; Kumar et al., 2017; Parikh et al., 2018). Diarrhea and nausea are the most common AEs of chemotherapy (Gralla et al., 1981) and so as venetoclax, although they are generally mild and few that appear as severe AEs. Except for anemia, the occurrence rates of grade 3 or 4 febrile neutropenia and thrombocytopenia are under 0.1. The occurrence rate of anemia in R/R CLL patients was significantly higher than untreated when venetoclax combined with obinutuzumab (Cramer et al., 2018; Flinn et al., 2019), and the reason needs further exploration.

About 40% patients experienced neutropenia after taking venetoclax, and most of them went through grade 3 or 4 AE. Fortunately, none of these patients quit the treatment because of severe neutropenia. Most patients with neutropenia received growth factor or reduced the venetoclax dose (Wierda et al., 2005; Robak et al., 2010). It is also suggested that patients receive intermittent treatment with neutrophil growth factor since this method enabled continuous venetoclax treatment in majority of the patients with grade 4 neutropenia. Neutropenia and TLS were reported in only one study, while TLS is not severe and it could be relieved by growth factor (Seymour et al., 2017). Some researches reported that the occurrence of neutropenia in venetoclax monotherapy was consistent with previous chemotherapy or immunotherapies in patients with R/R CLL (Hallek et al., 2010). The rate of neutropenia in NHL patients was less than that in CLL patients, while severe AEs were more common (Davids et al., 2017). The condition of MM patients was similar to that of NHL patients (Kumar et al., 2017). In addition, neutropenia is rare in R/R AML patients (Konopleva et al., 2016).

In our research, the ORR of venetoclax monotherapy can reach 73%, while the combination of lenalidomide and rituximab used in R/R CLL patients was only 66%, and the combination of bendamustine and rituximab remained less effective. There are two involved articles that mentioned deletion of chromosome 17p (del[17p]) in patients with CLL. The past articles reported that these del[17p] patients had quite a bad prognosis and ORR. When patients received ibrutinib and idelalisib treatment, del[17p] was a high risk factor, leading patients discontinuation of treatment (Maddocks et al., 2015). The first trial of venetoclax showed that the response rate of the R/R CLL patients with deletion 17p was 71%; about 16% achieved a complete response (Roberts et al., 2016). In another study, all the involved patients were R/R CLL patients with deletion [17p]; the ORR reached 79.4% (Stilgenbauer et al., 2016). Venetoclax is worthy to be recommended to the CLL patients with deletion [17p]. Patients with MCL have the highest ORR (75%) of all the NHL patients, which is similar to the Bruton tyrosine kinase inhibitor ibrutinib (Wang et al., 2013). The ORRs of other NHL patients were more inferior compared with the MCL monotherapy patients. When venetoclax was combined with other therapies, the response rate of diffuse large B-cell lymphoma and follicular lymphoma was promising compared with historical treatment (Marcus et al., 2017; Vitolo et al., 2017). While the response rate of different subtypes was quite different, the response rate of venetoclax was also influenced by various combining methods. Survival rates of elderly patients with R/R AML were quite poor (Pinto et al., 2001). The ORRs of the R/R AML in elderly patients differed tremendously among the reported studies. In our studies, the ORR was 19% with oral monotherapy. It was reported that over 20% remission rates were rarely acquired without other intense cytotoxic chemotherapy and inpatient care (Breems et al., 2005; Thol et al., 2015). When venetoclax plus decitabine or 5-azacitidine was used in R/R AML patients, the ORR was over 60% (Aldoss et al., 2018). Venetoclax combined with other drugs was also used in untreated AML. To elderly patients, venetoclax plus low dose cytarabine gets more favorable results than decitabine and azacitidine monotherapy treatment relatively (Kantarjian et al., 2012; Dombret et al., 2015; Wei et al., 2019). The ORR of MM patients treated with venetoclax plus bortezomib and dexamethasone was satisfactory with tolerable AEs (Moreau et al., 2017). When venetoclax combined with obinutuzumab, the patients had a quite satisfactory ORR; nearly all the patients benefit from this method.

Compared with venetoclax monotherapy, venetoclax combined with other therapies did not obviously increase the rate of thrombocytopenia, neutropenia, and anemia, while the rates of peripheral edema and upper respiratory tract infection increased among patients applying two or more therapies. The ratio of grades 3 to 4 thrombocytopenia slightly increased in the patients using venetoclax plus decitabine. Thrombocytopenia had been reported as the most frequent severe AEs of decitabine (He et al., 2017). In conclusion, the combination with other therapies was also well tolerated. There was no significance between the monotherapy and combination groups.

In our study, we admit that several limitations are inevitable. The dose of the drug was different between individual trials, and some AEs were dose dependent. Some of the studies chose dose-escalation method to evaluate the characters of the drug. In addition, all the involved studies were single-armed studies without double-blinded randomized controlled trials. Lastly, studies rarely use the same treatment method; it is hard to say which one is better for the patients. More researches were expected. Although the patients are all in advanced stages, the degree of malignancy was dispersive, which might have aroused bias to the final analysis.

## Conclusion

In conclusion, venetoclax is a mild and efficient drug in treating advanced hematological malignancy specially fit for the CLL patients with del[17p]. Neutropenia is relatively severe among the patients, and it can be alleviated by reducing dose or some other ways. Few TLS occurred after taking the drugs. The AEs aroused by venetoclax and combination are well tolerable. The use of venetoclax monotherapy or its combination with other therapies is desirable in hematological malignancy.

## Contribution to the Field Statement

Venetoclax has shown effectiveness in the treatment of patients with hematological malignancy. However, toxicities and clinical benefits of venetoclax in cancer patients are not well defined. To systematically review the safety and efficacy of venetoclax in the treatment of patients, we retrieved all the relevant clinical trials on the adverse events (AEs) and survival outcomes of venetoclax through PubMed. Ten eligible studies involving a total of 726 patients met our meta-analysis criteria. After analysis of the data, we found that venetoclax is a mild and efficient drug in treating advanced hematological malignancy that is specially fit for the CLL patients with del[17p], and AEs are well tolerable. Few tumor lysis syndrome (TLS) occurred after taking the drugs. The AEs aroused by venetoclax and combination are well tolerable. Therefore, the use of venetoclax monotherapy or its combination with other therapies is desirable in hematological malignancy.

## Data Availability Statement

The datasets generated for this study are available on request to the corresponding author.

## Author Contributions 

QL and XM mainly generated the idea. QL and LC collected the data and wrote the manuscript. HJ and HL helped correct the grammar and carried out some statistical work. KS and QL mainly did some statistical work. YC helped in collecting the data.

## Conflict of Interest Statement

The authors declare that the research was conducted in the absence of any commercial or financial relationships that could be construed as a potential conflict of interest.
