# Supplementary material for: Efficacy and Safety of Bcl-2 Inhibitor Venetoclax in Hematological Malignancy: A Systematic Review and Meta-Analysis of Clinical Trials
Source: Front Pharmacol. 2019 Jun 21;10:697. doi: 10.3389/fphar.2019.00697 (PMC6598635; doi:10.3389/fphar.2019.00697)
Supplement: Supplementary file 1 [file Table_1.docx]

| Study | Treatment | Disease | ORR | Media OS | Media PFS |
| --- | --- | --- | --- | --- | --- |
| Philippe Moreau | venetoclax plus bortezomib and dexamethasone | Relapsed or Refractory Multiple Myeloma | 67% | NM | 9.5 months(5.7-10.4) |
| Prof John F Seymour | venetoclax plus rituximab | Relapsed or Refractory Chronic Lymphocytic Leukaemia | 82%* | NM | Not Reached |
| Courtney D DiNardo A | venetoclax plus decitabine | untreated Acute Myeloid Leukaemia | 65% | 15.2 months (8.0–NR) | NM |
| Courtney D DiNardo B | venetoclax plus azacitidine | untreated Acute Myeloid Leukaemia | 59% | 14.2 months (9.3–NR) | NM |
| Courtney D DiNardo C | venetoclax plus decitabine and posaconazole | untreated Acute Myeloid Leukaemia | 67% | NM | NM |
| Courtney D DiNardo |  | untreated Acute Myeloid Leukaemia | 64% | 12.3 months (5.2–NR) | NM |
| [Ibrahim Aldoss](https://www.ncbi.nlm.nih.gov/pubmed/?term=Aldoss I[Author]&cauthor=true&cauthor_uid=29545346) | venetoclax plus decitabine or 5-azacitidine | Relapsed or Refractory Acute Myeloid Leukaemia | 64% | NR | 8.9months(3.2-10.6) |
| Paula Cramer | venetoclax plus obinutuzumab | Chronic Lymphocytic Leukaemia | 95% | NR | NR |
| Andrew H.Wei | venetoclax plus cytarabine | untreated Acute Myeloid Leukaemia | 54%  (CR) | 10.1months(5.7-14.2 ) | NM |
| Kerry A. Rogers | venetoclax plus obinutuzumab and ibrutinib | Relapsed or Refractory Chronic Lymphocytic Leukaemia | 92% | NM | NM |
| S. de Vos | venetoclax plus bendamustine and rituximab | Relapsed or Refractory Non-Hodgkin Lymphoma | 65% | NR | 10.7months( 4.3-21.0) |
| Andrew D. Zelenetz | ventoclax plud R/G-CHOP | Non-Hodgkin Lymphoma | 87.5% | NR | NR |
| Ian W. Flinn | venetoclax plus obinutuzumab | Chronic Lymphocytic Leukaemia | 95% (R/R),100 (1L) | NM | NM |
| Arnon P.Kater | venetoclax plus rituximab | Relapsed or Refractory Chronic Lymphocytic Leukaemia | NM | NM | NM |

ORR= Overall Response Rate; OS= Overall Survival,;NM=Not Mention;PFS=Progression Free Survival;NR=Not Reached;*This article provide the rate of response,NR=Not reached,CR=complete remission.
