# Supplementary figures and images for: Efficacy and Safety of Bcl-2 Inhibitor Venetoclax in Hematological Malignancy: A Systematic Review and Meta-Analysis of Clinical Trials
Source: Front Pharmacol. 2019 Jun 21;10:697. doi: 10.3389/fphar.2019.00697 (PMC6598635; doi:10.3389/fphar.2019.00697)

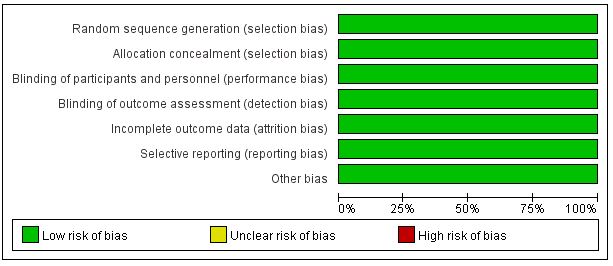

Supplement: Supplementary file 2 [file Image_1.jpeg]

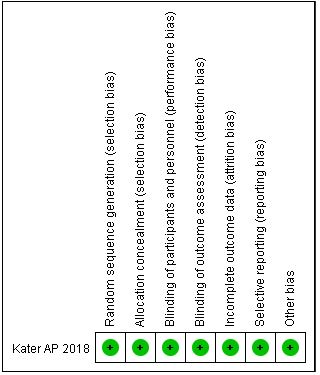

Supplement: Supplementary file 3 [file Image_2.jpeg]
